# CO_2_ Capture With Absorbents of Tertiary Amine Functionalized Nano–SiO_2_

**DOI:** 10.3389/fchem.2020.00146

**Published:** 2020-02-28

**Authors:** Nanjun Lai, Qingru Zhu, Dongyu Qiao, Ke Chen, Lei Tang, Dongdong Wang, Wei He, Yuemei Chen, Tong Yu

**Affiliations:** ^1^School of Chemistry and Chemical Engineering, Southwest Petroleum University, Chengdu, China; ^2^State Key Laboratory of Oil and Gas Geology and Exploitation, Chengdu University of Technology, Chengdu, China; ^3^State Key Laboratory of Polymer Molecular Engineering, Fudan University, Shanghai, China; ^4^Key Laboratory of Oilfield Chemistry (KLOC), CNPC, Beijing, China; ^5^Engineer Technology Research Institute, CNPC Xibu Drilling Engineering Company Limited, Ürümqi, China; ^6^China National Offshore Oil Corporation (CNOOC) Energy Development Company Limited, Tianjin, China

**Keywords:** nano–SiO_2_, tertiary amine, CO_2_ capture, low viscosity, regenerability

## Abstract

To improve CO_2_ adsorption performance of nanoparticle absorbents, a novel tertiary amine functionalized nano–SiO_2_ (NS–NR_2_) was synthesized based on the 3–aminopropyltrimethoxysilane (KH540) modified nano–SiO_2_ (NS–NH_2_) via methylation. The chemical structure and performances of the NS–NR_2_ were characterized through a series of experiments, which revealed that NS–NR_2_ can react with CO_2_ in water and nanofluid with low viscosity revealed better CO_2_ capture. The CO_2_ capture mechanism of NS–NR_2_ was studied by kinetic models. From the correlation coefficient, the pseudo second order model was found to fit well with the experiment data. The influencing factors were investigated, including temperature, dispersants, and cycling numbers. Results has shown the additional surfactant to greatly promote the CO_2_ adsorption performance of NS–NR_2_ because of the better dispersity of nanoparticles. This work proved that NS–NR_2_ yields low viscosity, high capacity for CO_2_ capture, and good regenerability in water. NS–NR_2_ with high CO_2_ capture will play a role in storing CO_2_ to enhanced oil recovery in CO_2_ flooding.

## Introduction

In recent years, humans have been endangered by greenhouse effect leading to global warming. Carbon dioxide (CO_2_) emission source from the burn of fossil fuels catches much attention because of it is a major factor to the greenhouse effect (Sarkodie and Strezov, 2019). One method to assuage the greenhouse effect is to capture CO_2_ from emission sources and then save it in stratum or apply it for enhanced oil recovery in low permeability reservoirs. Therefore, a feasible approach called carbon capture and storage (CCS) technologies have developed, including membranes, cryogenic distillation, gas adsorption with liquids or solids, and others (Benson and Orr, 2011; Bui et al., 2018). However, membrane–based separation is not a suitable way for CO_2_ capture because perfection wants to be made in the areas of CO_2_ selectivity, permeability, cost, and performance depletion over time (all caused by a variety of factors). Moreover, because of the high energy costs involved, cryogenic distillation is not optimally suitable. Solvents and solid sorbents have been reported for CO_2_ capture, such as basic solvents, supported amine, and ammonium materials, as the primary classes of chemical sorbents (Heydari-Gorji et al., 2011; Darunte et al., 2016; Hahn et al., 2016; Sanz-Perez et al., 2016; Kong et al., 2019). The adsorption of CO_2_ by solvents is a commercially available method, but the regeneration process of the solvents is highly energy intensive and expensive (Rochelle, 2009). The adsorption of CO_2_ by solid sorbents has recently attracted much attention in the study of CO_2_ capture (Lee and Park, 2015).

Nano silica (SiO_2_) solid sorbents have been investigated for CO_2_ capture given their high pore volume, large surface, and ease of functionality (Liu et al., 2016; Lai et al., 2017, 2019; Wang et al., 2019). Jiao et al. (2015) prepared mesoporous silica (MSU–J) with a wormhole framework texture, the surface of MSU–J was modified with triethylenetetramine (TETA) for CO_2_ capture. Hahn et al. (2016) reported the primary amine, secondary amine, and bibasic amine species functionalized porous SiO_2_ and discussed the mechanism of CO_2_ adsorption on the SiO_2_. Bae (2017) showed that the 3–(2–aminoethylamino) propyldimethoxymetylsilane modified silica can be used as an adsorbent to improve CO_2_ capture performance and obtained capture CO_2_ capacity of 2.24 mmol/g. The surface of SiO_2_ usually has been functionalized with primary and secondary amines for CO_2_ capture. Amine modified SiO_2_ reacted with CO_2_ to form carbamate or bicarbonate species based on the acid–based chemical interaction for improved CO_2_ adsorption (Huang et al., 2003). Without the presence of water, the amine groups reacted with CO_2_ molecules to create the carbamates group. As another specific, the presence of water impairs this amino group adsorption (Ma et al., 2017). Therefore, the functionalized nano–SiO_2_ with water impede is needed to further investigated in future development.

It is difficult to destroy the steady carbon–nitrogen bond of carbamate that is formed in primary amine and secondary amines reaction with CO_2_. Also, fascinating tertiary amines as absorbents generate bicarbonates to replace carbamates when tertiary amines reacting with CO_2_ (Crooks and Donnellan, 1990; Vaidya and Kenig, 2007) thereby leading to low energy for regeneration of absorbents. Therefore, tertiary amine as an energy saving absorbent is appropriate comparing with primary amines and secondary amines (Gao et al., 2017). Particularly, the rate of primary amines and secondary amines with CO_2_ is faster than tertiary amines (Liu et al., 2019a). However, the solubility of CO_2_ is higher in tertiary amines solution than one in primary amines and secondary amines solution due to different reaction mechanisms. For example, the reaction molar ratio of tertiary amine and CO_2_ is 1:1 to formed bicarbonate structure, while the reaction molar ratio of primary amine or secondary amine and CO_2_ is 0.5:1 to formed carbamate structure (Sartorl and Savage, 1983). Tertiary amine can be able to generate a bicarbonate due to no hydrogen on nitrogen when reacted with CO_2_ and H_2_O, resulting in a better CO_2_ adsorption and lower energy depletion for regeneration (Xiao et al., 2016, 2019). Moreover, kinetics is important since it can explain the dynamic adsorption of the sorbent, a lot of kinetic models are applied to the CO_2_ adsorption property of tertiary amine (Liu et al., 2019b).

Therefore, this study aimed to develop a sorbent to avert the limits of aqueous amine solutions and take advantage of tertiary amines for CO_2_ capture. The tertiary amine loaded nano–SiO_2_ was synthesized, and the CO_2_ capture performance was studied in the presence of water. The CO_2_ adsorption mechanism was investigated by kinetics, and the viscosity of the absorbent dispersion was measured before and after CO_2_ adsorption. Finally, tertiary amine functionalized nano–SiO_2_ (NS–NR_2_) was investigated further in terms of temperature, dispersants, and cycling numbers.

## Experimental Section

### Materials

Methylbenzene(C_7_H_8_), 3–(trimethoxysilyl)−1–propanamine (KH−540), ethanol (C_2_H_5_OH), formic acid (HCOOH), formaldehyde (HCHO), hydrochloric acid (HCl), N, N–dimethylformamide (DMF), and sodium hydroxide (NaOH) were purchased from Chengdu Kelong Chemical Reagent Factory (Sichuan, China). Nano–SiO_2_ (10–20 nm) was obtained from Aladdin Chemistry Co. (Shanghai, China). All chemical reagents were analytical–grade. CO_2_ (g) and N_2_ (g) were purchased from Jingli Gas Company (Chengdu, China). Water was double deionized with a Millipore Milli Q system to produce the 18 MM deionized water.

### Synthesis and Characterization of Tertiary Amine Functionalized Nano–SiO_2_

**Figure 1:**
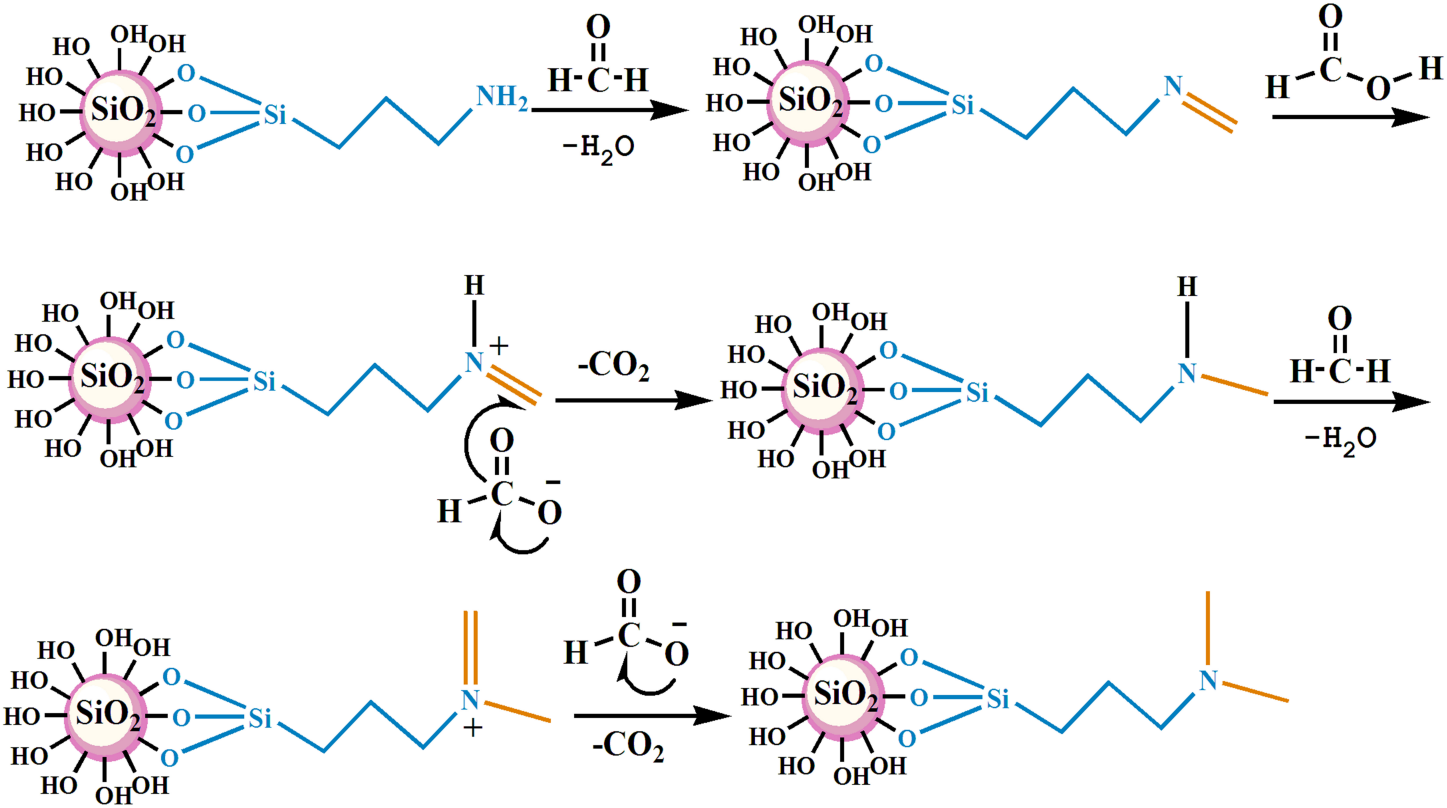
Schematic of the reaction steps of NS–NR_2_.

The nano–SiO_2_ loaded with primary amines (NS–NH_2_) was prepared first using 3–Aminopropyltrimethoxysilane (KH540) as modifiers, and then it was used as the matrix material to synthesize branched nanomaterials with a tertiary amine group on its surface (NS–NR_2_) via methylation of primary amines based on formic acid and formaldehyde. The mechanism is shown in Figure 1.

The specific reaction conditions of NS–NH_2_, NS–NR_2_, and the preparation methods of the nanofluid are shown in Supplementary Materials. It is worth noting that add anhydride to promote unreacted primary amine groups undergo acetylation. The optimum reaction conditions of NS–NR_2_ are displayed in Table 1.

**Table 1:** Conditions of NS–NR_2_.

**Time (h)**	**Temperature (^**°**^C)**	**NS-NH_**2**_: HCOOH: HCHO (g:mol:mol)**	**Solvent (mL)**
12	90	1:6:6	60

Fourier transform–Infrared (FT–IR) spectra were acquired by the KBr pellet method using a WQF520 spectrometer. Thermogravimetric analysis (TGA) was conducted on a synchronous comprehensive thermal analyzer (Netzsch Scientific Instruments). The microtopography of NS–NR_2_ was characterized using an electron microscope (ZEISS Libra 200 FE). The carbon and nitrogen contents were detected by elemental analysis using a Var10EL III instrument. The hydrodynamic diameter and proportion of the nanoparticles were received by a BI 200SM wide-angle dynamic light scattering (DLS) instrument. The rheological property of NS–NR_2_ dispersion was measured with a HAAKE MARS III rheometer at 25°C to assess the viscosity.

### CO_2_ Adsorption and Desorption

NS–NR_2_ dispersion was introduced into a gas adsorption bottle. The gas adsorption bottle was put in a constant temperature water bath. The gas flow of CO_2_ was controlled at 1 L/min by a gas flow controller, and CO_2_ was bubbling into the high concentrated sulfuric acid to adsorb microscale water in CO_2_ gas in a hermetic wild-mouth bottle. After that, the dry CO_2_ was flowed through the gas adsorption bottle to reacted with absorbent in the water. The mass change of dispersion was confirmed by an accurate analytical balance (±0.1 mg) until the weight has no change. The amount of adsorption CO_2_ on nanoparticles could be calculated under a control test (no nanoparticles). As shown in Figure S1, most of the tests were implemented to assure repeatability of the method. The CO_2_ adsorption capacity of NS–NR_2_ was measured using a gas adsorption system, as shown in Figure S2. The CO_2_ desorption experiment was simply carried out by bubbling N_2_ around 1 L/min instead of injecting CO_2_ and keeping the system temperature at 25°C to avoid the huge energy depletion.

The mechanism studies of CO_2_ adsorption into NS–NR_2_ are often executed using kinetic models including pseudo first order, pseudo second order, and intraparticle diffusion model. The parameter *R*^2^ and relative error (ε) were applied to evaluate the reliability of kinetic models in predicting adsorption capacity, as defined in Equation (1). Based on Equation (1), *q*_*e, cal*_ is the predicted date acquired from the model analysis, and *q*_*e, exp*_ is the experiment date.

(1) $$\begin{array}{l} \varepsilon \left(\%\right)=\frac{q_{ecal}-q_{eexp}}{q_{eexp}}\times 100\% \end{array}$$

The pseudo first order model that introduced by Langergren in 1898 year (Langergren, 1898) is shown in Equation (2):

(2) $$\begin{array}{l} \log\left(q_{e}-q_{t}\right)=\log\left(q_{e}\right)-\left(\frac{k_{1}}{2.303}\right)t \end{array}$$

where *q*_*t*_ is the adsorption capacity at a special time, *q*_*e*_ is the adsorption capacity at equilibrium, *k*_1_ is the constant of pseudo first order with a unit of 1/min. The pseudo second order model (Ho and McKay, 1999) is represented by Equations (3) and (4):

(3) $$\begin{array}{l} \frac{t}{q_{t}}=\frac{1}{k_{2}q_{e}^{2}}+\frac{t}{q_{e}} \end{array}$$

(4) $$\begin{array}{l} h=k_{2}\cdot q_{e}^{2} \end{array}$$

where *q*_*t*_ is the adsorption capacity at a special time, *q*_*e*_ is the adsorption capacity at equilibrium, *k*_2_ is the constant of pseudo second order with a unit of g/mmol min. The intraparticle diffusion model offers the diffusion mechanism of matter in adsorption process as defined in Equation (5):

(5) $$\begin{array}{l} q_{t}=kt^{1/2}+C \end{array}$$

Where *q*_*t*_ is the adsorption capacity at a special time (mmol/g), *k* is the rate constant of intraparticle diffusion (mmol/g min^1/2^) and C (mmol/g) is as the thickness of the boundary layer; the intercept is positively correlated with the boundary layer. (Hameed et al., 2008; Yousef et al., 2011). Mass transfer of adsorbate to the adsorbent surface (bulk diffusion) and film diffusion into the internal sites (intraparticle diffusion) and other steps occur in the process of adsorption.

## Results and Discussion

### Characterization

**Figure 2:**
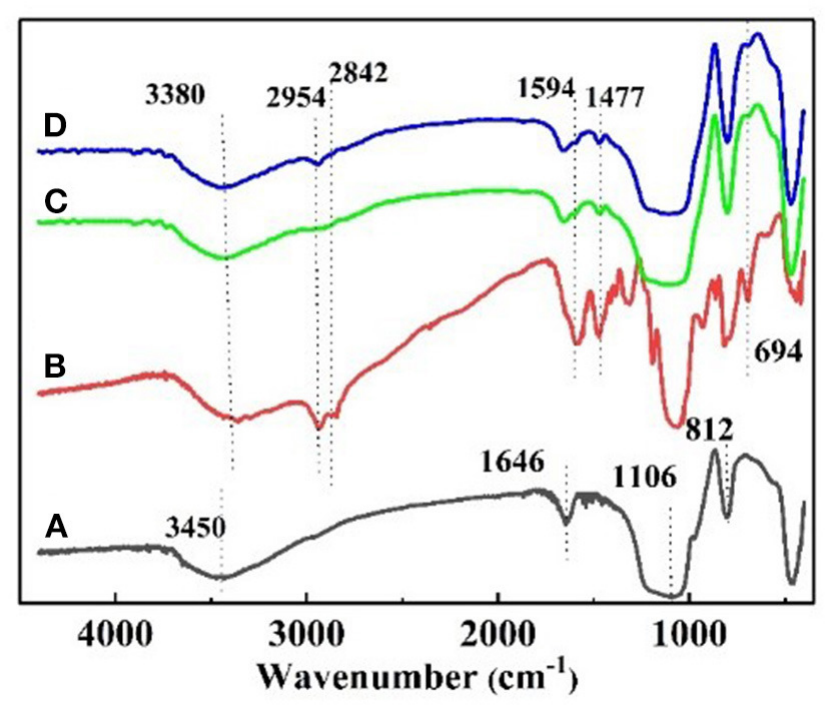
**(A)** FT–IR spectra of raw SiO_2_, **(B)** KH540, **(C)** NS–NH_2_, and **(D)** NS–NR_2_.

The FT–IR spectrum of the nanoparticles is shown in Figure 2. Figure 2A shows the strong adsorption peaks at around 3,450 and 1,646 cm^−1^, suggest the stretching vibration of the –O–H bonds on the surface of silica. The adsorption peaks near 1,106 and 812 cm^−1^ are the adsorption peaks of the Si–O–Si group, which are characteristic adsorption peaks of SiO_2_. In the FT–IR spectra of KH540, the adsorption peaks around 3,380 and 1,594 cm^−1^ are the –N–H stretching and NH_2_ deformation of hydrogen bonded amino groups (Jiao et al., 2015). The adsorption peak at 1,477 cm^−1^ is C–N, and the peaks at 2,954 and 2,842 cm^−1^ are feature adsorption peak of –CH_3_ and –CH_2_-, respectively. The peak at 694 cm^−1^ is the adsorption peak of Si–C (Titinchi et al., 2014). In the FT–IR spectra of NS–NH_2_, the 2,954 cm^−1^ peak of –CH_3_ disappeared, elucidating that the primary amine was grafted on the surface of SiO_2_. In Figure 2D, the adsorption peak of –CH_3_ is shown to appear, indicating that –(CH_2_)_3_NH_2_ reacted to –(CH_2_)_3_N(CH_3_)_2_ on the surface of nano–SiO_2_.

**Figure 3:**
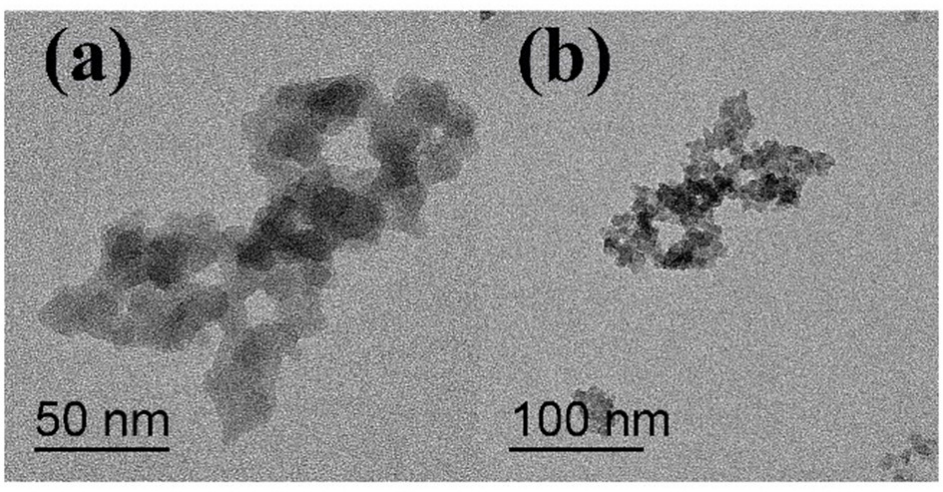
TEM image of NS–NR_2_ (the scale bars in **a** is 50 nm and **b** is 100 nm).

**Figure 4:**
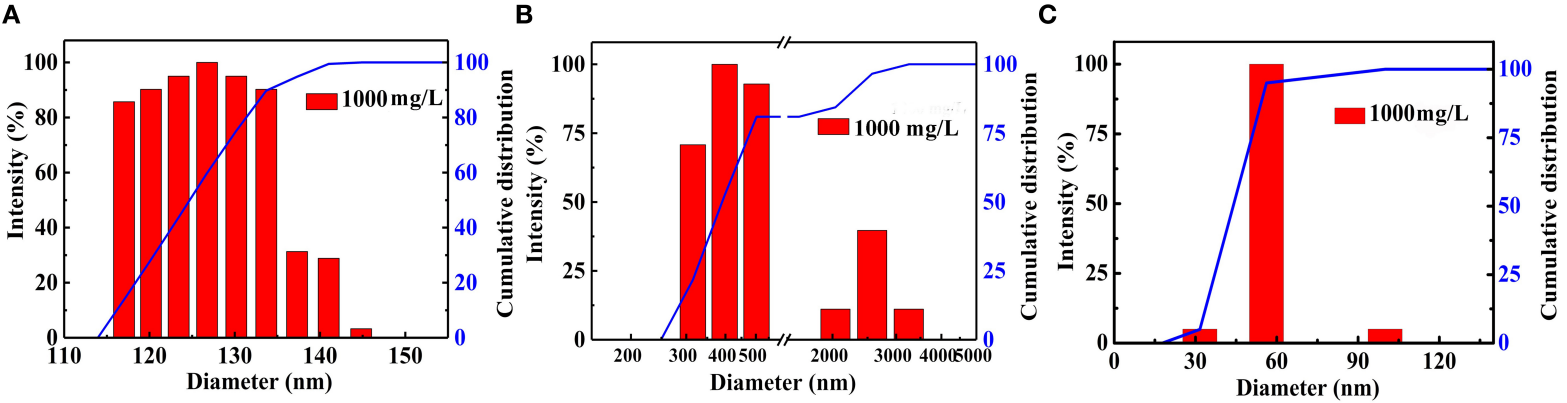
Diameter distribution of **(A)** NS–NR_2_ and **(B)** nude nanoparticles in water; **(C)** Diameter distribution of NS–NR_2_ in PEG−400 solution (1,000 mg/L).

The microscopic structure of the nanoparticles is shown in Figure 3 as observed from the TEM morphology. The diameter of NS–NR_2_ was shown to be approximately 15 nm. The nanoparticles aggregated slightly because of the particle size being in the nanometer scale (Zhao et al., 2014). The evidence from DLS analysis (Figures 4A,B) shown the hydrodynamic diameter of NS–NR_2_ to be approximately 123.6 nm with uniform size distribution. Moreover, it was found that the diameter distribution of nude particles was wider and the agglomeration was more serious than that of the modified nanoparticles.

The element contents of raw SiO_2_, NS–NH_2_, and NS–NR_2_ are shown in Table 2. Carbon and nitrogen contents are present in raw SiO_2_ because many SiO_2_ materials are often synthesized in an aqueous organic solvent, resulting in remaining carbon and nitrogen in such materials. The contents of carbon and nitrogen for NS–NH_2_ were shown to be 5.22 and 1.65 mmol/g, respectively, and for NS–NR_2_ were shown to be 7.14 and 1.39 mmol/g, respectively. The contents of these elements were much higher than raw SiO_2_. The nitrogen amount in NS–NR_2_ was lower than that of NS–NH_2_ because when the molar amount of carbon increases, the molar amount of nitrogen decreases in fixed mass products. The molar ratio of carbon to nitrogen is 3.16, 5.13 in NS–NH_2_ (SiO_2_-C_3_H_8_N) and NS–NR_2_ (SiO_2_-C_5_H_10_N), respectively. Here, the molar ratio of carbon to nitrogen was adopted to further confirm the grafting of tertiary amines on nano–SiO_2_.

**Table 2:** Element contents on nanoparticles.

**Sample**	**C (mmol/g)**	**N (mmol/g)**	**Molar ratio of C/N**
Raw SiO_2_	0.15	0.18	0.85
NS-NH_2_	5.22	1.65	3.16
NS-NR_2_	7.14	1.39	5.13

**Figure 5:**
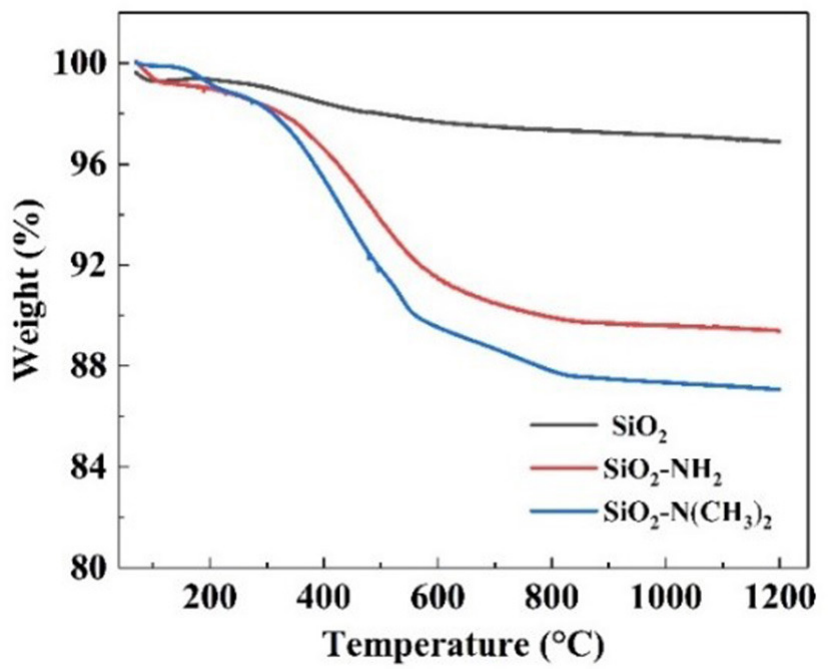
TGA thermograms of raw nano–SiO_2_, NS–NH_2_, and NS–NR_2_.

The successful synthesis of NS–NR_2_ could also be proved by thermos gravimetric analysis (TGA). Based on the TGA curves in Figure 5, the weight retention of raw nano-SiO_2_, NS–NH_2_ and NS–NR_2_ at 900°C under the air atmosphere were 96.86, 89.38, and 87.06%, respectively. For raw nano-SiO_2_, the mass depletion is attributed to the surface dihydroxylation. In terms of the structure of NS–NH_2_ and NS–NR_2_, when the temperature up to 900°C, the primary amine and tertiary amine chains were grafted on the nano-SiO_2_ has decomposed, respectively. Therefore, compared with the TGA curve of raw nano-SiO_2_, the surface of nano-SiO_2_ was modified to primary amine. The different weight loss between in the NS–NH_2_ and NS–NR_2_ indicated that tertiary amine was synthesized from primary amine successfully.

### CO_2_ Adsorption and Kinetic Studies

Any amine with a pKa value >5 can react with CO_2_ in the presence of water (Field and Grolimund, 1988). According to Figure S3A, the pH value of NS–NR_2_ dispersion decreased gradually with the addition of diluted hydrochloric acid (0.01 mol/L). The second derivative of the V_HCl_-pH curve was obtained (Figure S3B), with the zero point of the second derivative corresponding to the extreme point of the curve in Figure S3A. As a result, the pKa value was 7.08. The pKa value of 7.08 indicates that NS–NR_2_ dispersion can complete adsorption of CO_2_. Along with the aeration of CO_2_ at 1 L/min, the pH value of NS–NR_2_ dispersion dropped rapidly in the first 5 min, after which there were no changes in pH value for 20 min, as shown in Figure S3C, which means that NS–NR_2_ dispersion (0.1 wt.%) completely saturated CO_2_ at 1 L/min CO_2_ gas velocity in 20 min.

**Figure 6:**
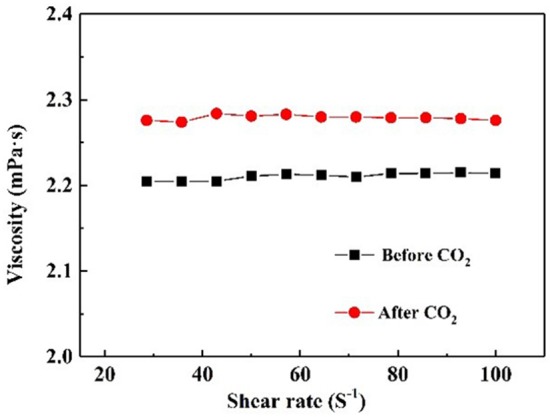
Viscosity of NS–NR_2_ dispersion before and after adsorption of CO_2_ as a function of shear rate.

**Figure 7:**
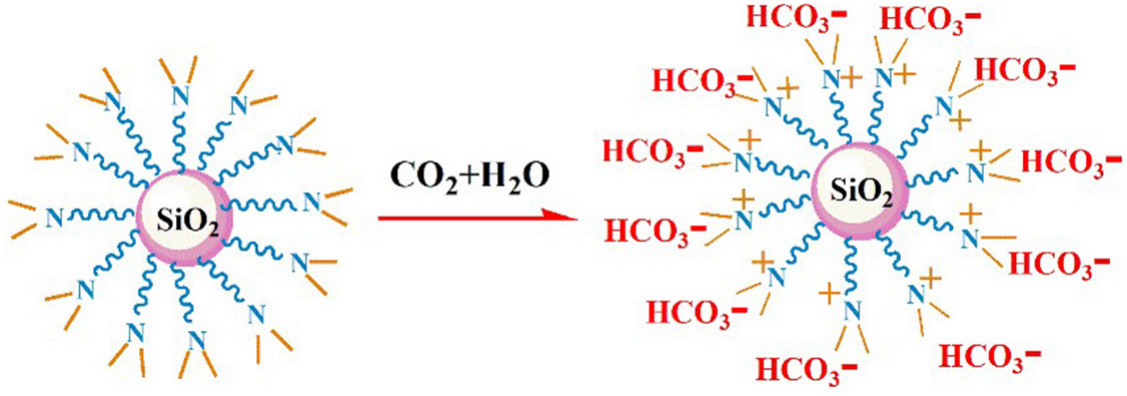
The process for NS–NR_2_ reacting with CO_2_.

It is well-known that CO_2_ capture is significant influenced by the viscosity of absorbent (Xiao et al., 2019). Figure 6 demonstrates the rheological property of NS–NR_2_ dispersion before and after adsorption CO_2_. A rheological plateau in the shear rate region was found for NS–NR_2_ dispersion. Before CO_2_ adsorption, the nanofluid viscosity was 2.23 mPa·s. After saturation with CO_2_, there was a nanofluid viscosity increase to 2.96 mPa·s. This viscosity change is consistent with the ionic liquids in Xiao et al.'s work (Xiao et al., 2019). A possible conclusion is the increase of electrostatic interaction of chains on the NS–NR_2_ surface due to CO_2_ adsorption (Figure 7), resulting in the higher viscosity. However, unlike the high viscosity of ionic liquids, the nanofluid viscosity was very low. The CO_2_ capture was not influenced by the increased viscosity.

The CO_2_ adsorption capacity of NS–NR_2_ was 25 mmol CO_2_/g NS–NR_2_ in water (0.1 wt.%) at 25°C, as shown in Table 3. Compared with other materials (with CO_2_ adsorption capacity of 0.1–21.45 mmol/g) (Yu et al., 2012), NS–NR_2_ in water has a better CO_2_ adsorption capacity.

**Table 3:** CO_2_ adsorption of samples at 25°C.

**Samples**	**Volume**	**CO_**2**_ adsorption**
Nanofluid (NS-NR_2_ of 0.06g)	60mL	5.55mmol
Water	60mL	4.05mmol

**Figure 8:**
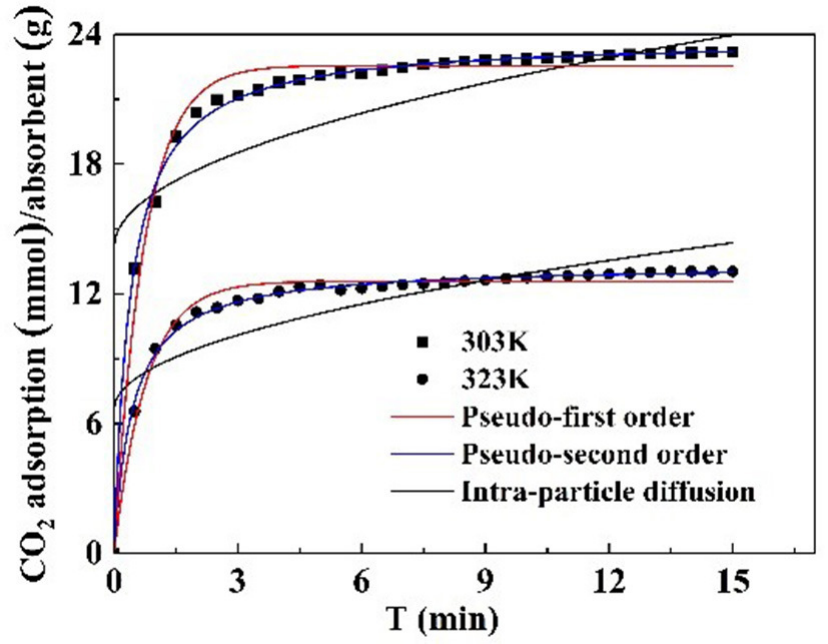
Kinetic plot of CO_2_ adsorption models at various temperatures.

Kinetic models, such as pseudo first order, pseudo second order, and intraparticle diffusion model, were applied to model the test data. The pseudo first order model is mostly appropriated to describe purely physisorption process without considering the any chemical reaction between CO_2_ molecules and sorbent. The pseudo second order model is mostly appropriated to describe purely chemisorption processes with stable chemical bonds between CO_2_ molecules and the sorbent. The comparison between the test curves and simulative curves are shown in Figure 8. The model parameters of kinetic at different temperatures and their corresponding coefficients are shown in Table 4. The *R*^2^ parameters of pseudo second order and pseudo first order models with experimental data were 0.99 and 0.97, respectively. The relative error for the pseudo second order model, ε, was lower than pseudo first order model. Compared with the *R*^2^, ε parameters, the pseudo second order model was found to fit well with the experiment data. Therefore, chemisorption of CO_2_ on nanoparticles plays a dominant role in CO_2_ capture.

**Table 4:** Kinetic parameters of CO_2_ adsorption on NS-NR_2_.

**Kinetic model**	**Parameter**	**Temperature (**^****°****^**C)**	
		**30**	**50**
	q_e,exp_ (mmol/g)	23.16	13.03
Pseudo first order	q_e,cal_ (mmol/g)	22.49	12.58
	K_1_ (1/min)	3.16	2.84
	R^2^	0.9795	0.9777
	Relative error, ε (%)	2.89	3.45
Pseudo second order	q_e,cal_ (mmol/g)	23.82	13.37
	K_2_ (g/mmol min)	0.10	0.17
	h (mmol/g min)	56.74	30.39
	R^2^	0.9977	0.9963
	Relative error, ε (%)	2.84	2.61
Intra-particles diffusion	K (mmol/g min^1/2^)	2.54	1.99
	C(mmol/g)	14.13	6.64
	R^2^	0.5044	0.6011

The pseudo first order model and pseudo second order model provide interaction insight into the actual mechanism of CO_2_ adsorption. The surface of the particles is grafted with functional groups to adsorb CO_2_. This surface adsorption was further verified by the intraparticle diffusion. The model parameters of intraparticle diffusion are revealed in Table 4. It is notable that no linear curve can pass through the origin point that is thought to be caused by intraparticle diffusion, suggesting that intraparticle diffusion is not the only factor controlling CO_2_ adsorption rate at all tested temperatures (Rashidi et al., 2013). Therefore, the adsorption process is not completely controlled by intraparticle diffusion, surface diffusion also plays a role in the whole CO_2_ adsorption process.

### Effect of Some Factors on CO_2_ Adsorption With NS–NR_2_

#### Effect of Temperature

**Figure 9:**
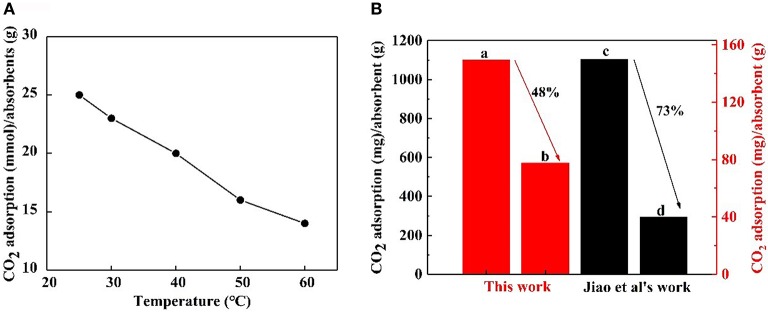
**(A)** CO_2_ adsorption of NS-NR_2_ in water at different temperatures. **(B)** CO_2_ adsorption of NS–NR_2_ at **(A)** 25°C and **(B)** 60°C; CO_2_ adsorption of MSU–J at **(C)** 25°C and **(D)**55°C.

A CO_2_ capture test was implemented using 0.1 wt.% nanofluid at contrast temperatures of 25, 30, 40, 50, and 60°C, respectively. The CO_2_ adsorption- temperature curve is revealed in Figure 9A. It is obvious that the maximum CO_2_ loading on the nanoparticles decreased at higher temperatures. Higher temperatures go against CO_2_ adsorption that is an exothermic reaction. The CO_2_ adsorption of NS–NR_2_ compared with that of MSU–J modified with TETA (Jiao et al., 2015) at higher temperatures is displayed in Figure 9B. The relative comparison presents a view of the advantages of the proposed nanoparticles although a slight difference in the experimental conditions. For the NS–NR_2_ absorbent in this work, the CO_2_ loading at 60°C decreased to 48% of the CO_2_ loading at 25°C, while the CO_2_ loading of MSU–J at 55°C decreased to 73% of the CO_2_ loading at 25°C. This result can be ascribed to the different mechanisms of CO_2_ adsorption. For modified MSU–J absorbent, huge energy was used to generate carbamate group with CO_2_. But adsorption of CO_2_ loaded nanoparticles enabled bicarbonate formation much easier because of the physical adsorption and chemical adsorption. This result indicates that the NS–NR_2_ in this work can capture more CO_2_ at higher temperatures.

#### Effect of Dispersants

**Figure 10:**
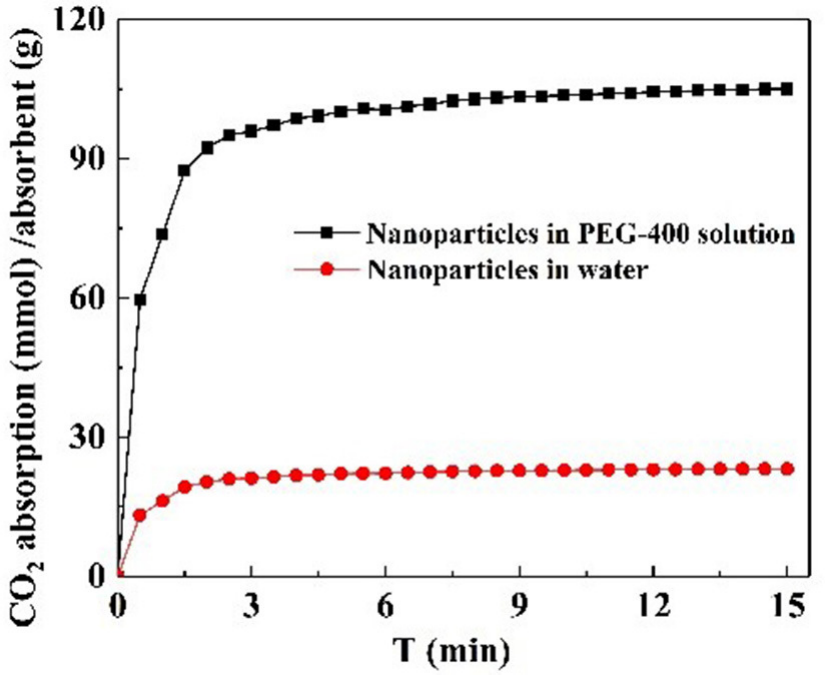
CO_2_ adsorption of NS-NR_2_ in different dispersants.

CO_2_ adsorption capacity in different dispersant agents were investigated. According to the previous test is shown in Figure S3C, it was not necessary to perform the experiment for longer than 20 min. Hence, CO_2_ adsorption with NS–NR_2_ was performed at a temperature of 30°C in different dispersants during this period of 15 min. CO_2_ adsorption curves are drawn in Figure 10. The maximum CO_2_ loading of NS–NR_2_ was changed in water or PEG−400 dispersants. The maximum CO_2_ loadings of NS–NR_2_ in water and PEG−400 solution was 23.16 and 105.34 mmol/g, respectively. This result can be explained by the diameter distribution of nanoparticles, where the diameter distribution in water was 123.6 nm and in PEG−400 solution was 56.7 nm (Figure 4C). The tertiary amine groups as specific sites for CO_2_ adsorption grafted on the surface of nano–SiO_2_ to endow the adsorbents with CO_2_ adsorption. Therefore, the more specific sites were exposed on the surface of nanoparticles because of better dispersibility. The dispersibility of nanoparticles is certified by the Derjaguin Landau Verwey Overbeek (DLVO) theory on interparticle interaction potential (Ilyas et al., 2014). The interaction among nanoparticles is caused by electrostatic repulsion and steric resistance in the PEG−400 solution. In addition, using PEG−400 as the dispersant is inexpensive and has lower surfactivity. Surfactants with similar properties can also be used to disperse the nanoparticles for CO_2_ capture. Therefore, adding the surfactant improved CO_2_ adsorption by enhancing the dispersibility of nanoparticles.

#### Effect of Cycling Numbers

**Figure 11:**
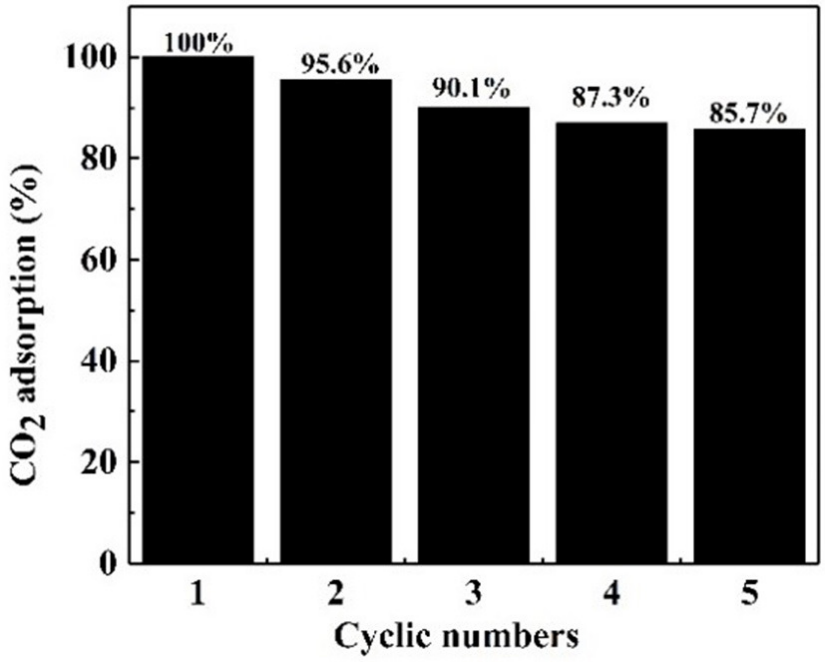
Cyclicity of NS–NR_2_ adsorbent in water.

It is crucial that an absorbent is reusable and retains an efficient CO_2_ adsorption capacity (Ma et al., 2017). Therefore, the regeneration tests of the NS–NR_2_ absorbent was carried out at 25°C. After CO_2_ adsorption in the nanofluid for 20 min, the nanofluid was shifted to CO_2_ desorption for regeneration. The CO_2_ desorption test was executed for another 20 min by bubbling N_2_ around 1 L/min before the absorbent was used for the next round of adsorption. As illustrated in Figure 11, five cycles of adsorption were implemented and the initial CO_2_ adsorption of absorbent was set as the 100 % baseline. After five cycles, NS–NR_2_ adsorbent shown favorable regeneration capacity with a slight decrease of 14.3% in water for NS–NR_2_ compared to the initial capacity. The results shown regeneration and efficient CO_2_ adsorption capacity of nanoparticles adsorbent. Considering cycling capacity, NS–NR_2_ material shows intriguing regeneration ability for CO_2_ adsorption.

## Conclusion

This work synthesized tertiary amino functionalized nano–SiO_2_ successfully. The measurements of pKa value and nanofluid viscosity change proved that NS-NR_2_ can react with CO_2_ in water and nanofluid has a low viscosity. NS–NR_2_ shown better CO_2_ adsorption capacity, and adsorption kinetics revealed the pseudo second order model was found to fit well with the experiment data. The influence of factors such as temperature, dispersants, and cycling numbers on CO_2_ adsorption was investigated. Results indicated higher temperature to work against CO_2_ adsorption of NS–NR_2_. The CO_2_ adsorption performance of NS–NR_2_ was greatly promoted because of a better dispersity of nanoparticles with added surfactant. After recycling of absorbent, the NS–NR_2_ maintained an efficient CO_2_ capture and shown favorable regeneration capacity. The measurements of NS–NR_2_ properties on the bases of viscosity, kinetic models, CO_2_ capture, and regeneration manifests that NS–NR_2_ exhibits satisfying performance to capture CO_2_. NS–NR_2_ with high CO_2_ capture will play a role in storing CO_2_ to enhanced oil recovery in CO_2_ flooding.

## Data Availability Statement

All datasets generated for this study are included in the article/Supplementary Material.

## Author Contributions

NL, QZ, and LT conceived the idea. QZ, WH, TY, and YC implemented the preparation, characterization, and measurement of NS–NR_2_. QZ wrote the manuscript. DQ and KC came up with ideas for the manuscript. QZ, LT, and DW discussed and analyzed the experiment data. LT and DW revised the manuscript. NL supervised the whole research work.

### Conflict of Interest

DQ was employed by the Engineer Technology Research Institute, CNPC Xibu Drilling Engineering Company Limited company. The remaining authors declare that the research was conducted in the absence of any commercial or financial relationships that could be construed as a potential conflict of interest.
