# Supplementary material for: CO2 Capture With Absorbents of Tertiary Amine Functionalized Nano–SiO2
Source: Front Chem. 2020 Feb 28;8:146. doi: 10.3389/fchem.2020.00146 (PMC7059254; doi:10.3389/fchem.2020.00146)
Supplement: Supplementary file 1 [file Table_1.DOCX]

**Supplementary Materials**

**CO_2_ Capture with Absorbents of Tertiary Amine–Functionalized Nano–SiO_2_**

Nanjun Lai, ^1,2,3,4,^* Qingru Zhu, ^1,4^ Dongyu Qiao, ^5^ Ke Chen, ^6^ Lei Tang, ^1,4^ Dongdong Wang, ^1,4^ Wei He, ^1^ Yuemei Chen, ^1^ and Tong Yu ^1^

*^1^**School of Chemistry and Chemical Engineering**, Southwest Petroleum University, Chengdu 610500, P. R. China*

*^2^**State Key Laboratory of Oil and Gas Geology and Exploitation, Chengdu University of Technology, Chengdu 610059, P. R. China*

*^3^**State Key Laboratory of Polymer Molecular Engineering, Fudan University, Shanghai 200438, P. R. China*

*^4^ Key Laboratory of Oilfield Chemistry (KLOC), CNPC, Beijing 100083 P. R. China*

*^5^ Engineer Technology Research Institute, CNPC Xibu Drilling Engineering Company Limited, Urumqi 830001, P. R. China*

*^6^ China National Offshore Oil Corporation (CNOOC) Energy Development Company Limited, Tianjin 300452, P. R. China*

**Correspondence: lainanjun@126.com; Tel.: +86-13094484238*

**Surface modification of nano-SiO_2_ (NS–NH_2_)**

5g SiO_2_ nanoparticles was weighed into a 250 mL three**–**necked flask followed by adding 80 mL methylbenzene as a solvent, then 2500μL KH540 was dispersed in the methylbenzene solution until homogeneity to react via constantly stirred and refluxed at 80 °C for about 12 h. Subsequently, the mixture was cooled to room temperature and was treated through vacuum filtering and washed at least three times with ethanol until all residues were removed. The solid product was dried at 80 °C for 24 h in a drying oven. The yield rate of modified nanoparticles is calculated by the potentiometric titration method. Crystal violet was used as an indicator and perchloric acid**–**glacial acetic acid was used as the standard liquid. Potentiometric titration is described as: in the process of titration, the abrupt change point of the color is usually end point of titration, but the abrupt change point of the color is not easy to determine. Therefore, the potential change and color of solution are recorded in the process of titration at the same time, the obtained potentiometric titration curve is differentiated by second order, and the point in the second order differential curve whose x-coordinate is zero is the end point of titration, in this way, the titration endpoint can be accurately determined the end point of titration.

0.2 g NS-NH_2_ was evenly dispersed in 20mL glacial acetic acid solution, two drops of crystal violet indicator were added, then the perchloric acid glacial acetic acid standard solution was quantitatively added for titration, and the potential of the dispersed solution was determined at the same time. The standard solution reacts with the primary amino group on the product surface as follows:


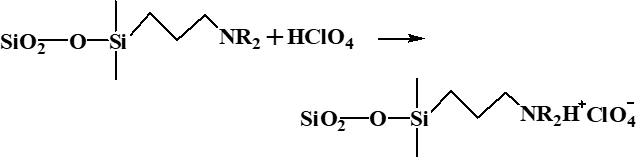


**Synthesis of NS–NR_2_**

1g Nano-silica with surface modification was scattered in 20**–**60 mL of DMF with ultrasonic treatment in a round bottom flask of 250 mL. While stirring, an appropriate amount of formic acid and formaldehyde (mol:mol=5**–**6:6) were introduced via constantly stirred and refluxed at 90**–**100 °C for about 6**–**18 h. After the reaction, the mixture was cooled to room temperature, then using vacuum filtering washed at least three times with ethanol until all residues were removed. Finally, the product was obtained by drying at 80 °C for 24 h in a drying oven. Crystal violet was used as an indicator and perchloric acid-glacial acetic acid was used as the standard solution. It is worth noting that add anhydride to promote unreacted primary amine groups undergo acetylation.

0.2g NS-NR_2_ was uniformly dispersed in 20mL glacial acetic acid solution, then 1.5 ml acetic anhydride was added to acetyl the unreacted primary amine groups into imines to eliminate the effects of primary amine groups. The reaction was as follows:


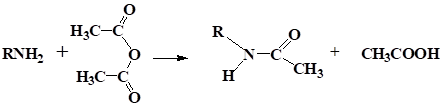


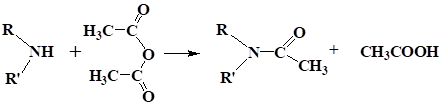


Two drops of crystal violet indicator were added, and the potential of the dispersed solution was determined at the same time. The standard solution reacts with the primary amino group on the product surface as follows:


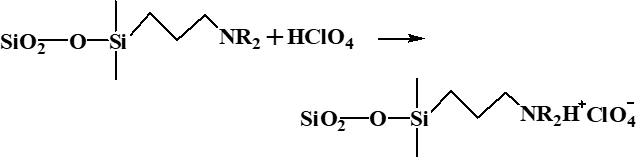


**Optimization of conditions**

The experiment conditions were optimized by four factors-three levels orthogonal factor (Table S1). The yield is calculated by the potentiometric titration method. Crystal violet was used as an indicator and perchloric acid**–**glacial acetic acid was used as the standard solution. It is worth noting that add anhydride to promote unreacted primary amine groups undergo acetylation.

**Table S1**. Orthogonal experimental design of synthesis NS**–**NR_2_.

| number | Time (h) | Temperature (ºC) | Ratio (mol) | Solvent (mL) | The yield of tertiary amine group (%) |
| --- | --- | --- | --- | --- | --- |
| 1 | 6 | 90 | 1:5:6 | 20 | 0.6289 |
| 2 | 6 | 95 | 1:6:6 | 40 | 0.7214 |
| 3 | 6 | 100 | 1:7:6 | 60 | 0.7666 |
| 4 | 12 | 90 | 1:6:6 | 60 | 0.7969 |
| 5 | 12 | 95 | 1:7:6 | 20 | 0.7124 |
| 6 | 12 | 100 | 1:5:6 | 40 | 0.7066 |
| 7 | 18 | 90 | 1:7:6 | 40 | 0.7508 |
| 8 | 18 | 95 | 1:5:6 | 60 | 0.7196 |
| 9 | 18 | 100 | 1:6:6 | 20 | 0.6738 |
| K1 | 0.9174 | 0.9432 | 0.8906 | 0.8733 |  |
| K2 | 0.9603 | 0.9332 | 0.9500 | 0.9442 |  |
| K3 | 0.9292 | 0.9305 | 0.9663 | 0.9894 |  |
| Range analysis | 0.0429 | 0.0128 | 0.0757 | 0.1161 |  |
|  | A2 | B1 | C3 | D3 |  |
|  | 12 | 90 | 1:7:6 | 60 |  |

Table S1 illustrated the reaction time was 12 h, temperature was 90 ºC, reactant ratio was 1:6:6 (NS-NH_2_: formic acid: formaldehyde), volume of solvent was 60 mL, which was the optimal reaction condition, and the most obvious factor influencing the reaction yield was the volume of solvent, secondly one was reactant ratio, the third one was reaction time, the last one was reaction temperature.


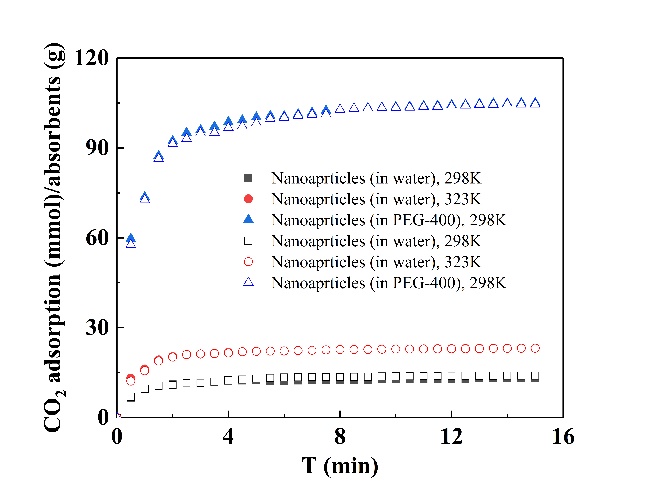


**Figure S1.** The repeatability of the CO_2_ absorption experiment. Solid: first experiments; Hollow: repeated experiments.


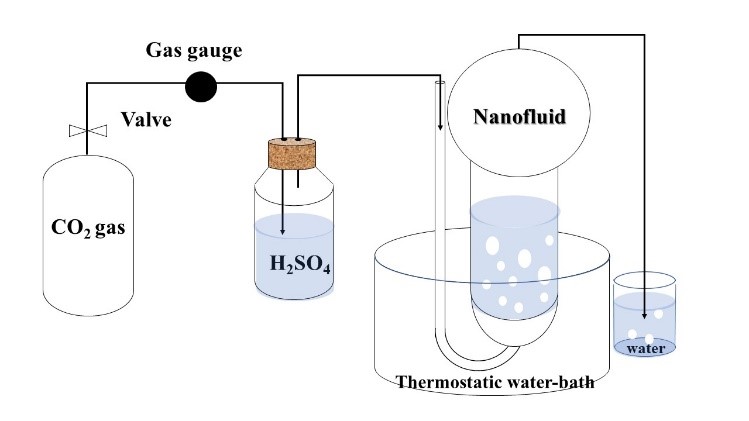


**Figure S2.** Schematic diagram for gas adsorption system.

**The preparation of NS-NH_2_/water dispersion**

100 mL of distilled water was added into a beaker, followed by the addition 0.1 g of NS**–**NR_2_, and then the suspension is dispersed by ultrasonic vibration for 10 minutes.

**The preparation of NS-NR_2_/PEG-400 dispersion**

The NS-NR_2_/PEG**–**400 dispersion (0.1 wt%) thus obtained were dispersed as follows. First, 100 mL of distilled water was added into a beaker, followed by the addition 0.03 g of PEG**–**400. Second, the solution was stirred for approximately 10 min in a water bath at 50 °C. Third, 0.1 g of NaOH was added into the solution. Next, 0.1 g of NS**–**NR_2_ was slowly added into the solution. After the temperature of the water bath was increased to 80 °C, the dispersion became clear and transparent for 15 min.


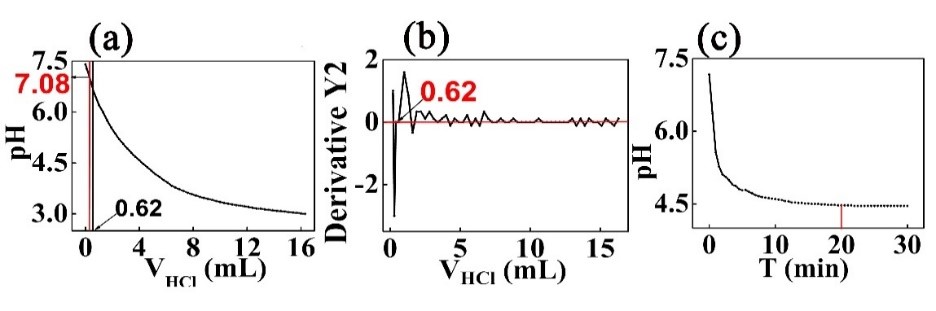


**Figure S3.** (a) V_HCl_–pH curve, (b) second–order differential of V_HCl_–pH curve, (c) pH variation with time.
